# Prolonged antibiotic treatment durations for community-onset infections in Dutch hospitals

**DOI:** 10.1017/ash.2026.10330

**Published:** 2026-03-27

**Authors:** Sharon E.J.D. van den Eijnde, Jesper van Breeschoten, Jurjen S. Kingma, Emile M. Kuck, Paul D. van der Linden, Cornelis H. van Werkhoven

**Affiliations:** 1 Julius Centre for Health Sciences and Primary Care, University Medical Centre Utrecht, Utrecht, Netherlands; 2 Department of Pharmacy, Tergooi Medical Centre, Hilversum, Netherlands; 3 Department of Pharmacy, Haga Hospital, The Hague, Netherlands; 4 Department of Clinical Pharmacy, Hospital Group Twente, Almelo and Hengelo, Netherlands; 5 Department of Hospital Pharmacy, Diakonessenhuis, Utrecht, Netherlands

## Abstract

**Background:**

Antibiotic stewardship programs (ASPs) aim to optimize antibiotic prescribing, as prolonged use increases the risk of adverse events, antimicrobial resistance, and unnecessary healthcare costs. This study aimed to determine the period prevalence of prolonged antibiotic treatment durations for community-onset infections in Dutch hospitals and to identify risk factors.

**Methods:**

A retrospective cohort study was conducted among adults treated for urinary tract infections (UTI), respiratory tract infections (RTI), or skin and soft tissue infections (SSTI) in four Dutch secondary care hospitals from January 1, 2021, to December 31, 2023. Patients were included if they were admitted for ≥12 hours and treated with antibiotics within 48 hours. Antibiotic prescriptions were linked to infectious diagnoses to calculate length of therapy and compared against national guidelines to assess prolonged treatment. Backward stepwise multivariable logistic regression was used to identify risk factors.

**Results:**

Of 9,878 admissions, 39.6% had UTIs, 44.9% RTIs, and 15.4% SSTIs. Prolonged antibiotic use was observed in 30%, with the highest proportion in RTIs (49.6%). Among RTIs, prolonged use occurred in 51.0% of community-acquired pneumonia (CAP) and 55.9% of aspiration pneumonia, with a combined median excess duration of 1.6 days (IQR: 0.9–3.6)). Prolonged use was 14.3% in UTIs and 13.1% in SSTIs. Risk factors included positive cultures, intravenous-to-oral switch, aspiration pneumonia or CAP (RTIs), and cystitis (UTIs).

**Conclusions:**

A high period prevalence of patients with RTIs receiving prolonged antibiotic treatment was observed. The identified risk factors should be considered in ASPs to improve prescribing and reduce non-guideline-concordant antibiotic therapy.

## Introduction

Antibiotic stewardship programs (ASPs) have been implemented to optimize antibiotic prescribing practices in hospitals, which includes promoting the shortest effective duration of antibiotic treatment.^1^ Excessive use of antibiotics is associated with an increased risk of adverse events, *Clostridioides difficile* infections, population-level multidrug-resistant pathogens, and unnecessary healthcare costs.^2–4^ However, a substantial proportion of antibiotic treatments initiated in hospitals exceed the recommended duration with data mainly from countries with higher antimicrobial resistance rates than the Netherlands.^4–7^ ASPs primarily focus on inpatient antibiotic prescribing practices,^8^ while patients also receive antibiotics upon discharge.

Commonly encountered infections in hospitals include respiratory tract infections (RTI), urinary tract infections (UTI), and skin and soft tissue infections (SSTI). For these infections, national treatment guidelines recommend antibiotic treatment durations ranging from five to fourteen days, depending on the specific clinical indication and patient factors.^9–13^ Although these infections account for a substantial proportion of overall antibiotic use, there is limited knowledge regarding current antibiotic prescribing practices and the treatment duration of these infections in Dutch secondary care hospitals. To determine the need for targeted antibiotic stewardship strategies, it is first necessary to identify patients who receive prolonged antibiotic treatment durations and to study factors contributing to it.

Therefore, the aim of this research is to determine the period prevalence of patients who received prolonged antibiotic treatment for community-onset RTI, UTI, or SSTI in Dutch hospitals. Additionally, to support future antibiotic stewardship activities, the study aims to identify risk factors associated with prolonged antibiotic treatment.

## Methods

### Study design and setting

A retrospective cohort study was conducted using data from four secondary care hospitals in the Netherlands (Tergooi Medical Centre (Hilversum), Haga hospital (the Hague), Hospital Group Twente (Almelo and Hengelo), and Diakonessenhuis (Utrecht)). This study was approved by the Institutional Research Board of Tergooi MC (study number 2024.54) and local approval was obtained at each of the participating hospitals. No consent procedure was required, as this was a retrospective study with pseudonymized data. Data was not collected from patients who, according to their medical records, had objected to the use of their data for research purposes.

### Study population

The study population consisted of patients who were hospitalized for at least 12 hours with a community-onset infection between January 1, 2021, and December 31, 2023, and who met all of the following inclusion criteria: age ≥18 years; an International Classification of Diseases and Related Health Problems-10 (ICD-10) diagnosis related to a RTI, UTI or SSTI (Supplement S1) during hospitalization; a discharge letter confirming a diagnosis of RTI, UTI or SSTI; and administration and prescription of the first systemic antibiotic dose within 48 hours of hospitalization. Patients were excluded if they met any of the following exclusion criteria: tested positive for, or were diagnosed with, COVID-19 within 48 hours before or after hospitalization (see Supplement S2 for definition), due to the risk of antibiotic overprescribing^14^; were transferred to or from another hospital, as treatment duration (length of therapy (LOT)) could not be reliably determined; had a concurrent infectious ICD-10 diagnosis during hospitalization (Supplement S1); clinical antibiotic LOT was below 0 days, which indicates a prescription error; antibiotic LOT exceeded 21 days, to exclude prophylactic antibiotic therapy; or died during hospitalization before completing the recommended duration of treatment, as they could not have received prolonged antibiotic therapy. Patients with empyema, for which only a minimal treatment duration is recommended,^15^ or with legionella infection, for which one local guideline recommends a total treatment duration of two to three weeks,^16^ making it impossible to distinguish between prolonged and chronic therapy, were also excluded from the RTI analysis.

Patients with a UTI were classified into a single category in the following order: catheter-associated UTI, UTI with systemic symptoms (eg, fever, delirium, flank pain), cystitis, or UTI—Other if none of the aforementioned criteria were documented in the patient’s electronic health record (EHR) (see Supplement S2 for definitions).^9,10^ Guideline adherence and prolonged treatment for UTI—Other were based on the cystitis treatment guidelines. Due to uncertainty about this classification, in an unplanned sensitivity analysis, they were instead based on treatment guidelines for UTI with system symptoms. Patients with a RTI were similarly classified in the following order: hospital-acquired pneumonia (HAP), aspiration pneumonia, severe community-acquired pneumonia (CAP-s) (defined as Pneumonia Severity Index (PSI) class 5 or CURB-65/AMBU-65 score of 3–5), mild-to-moderate community-acquired pneumonia (CAP-m) (PSI class 1–4 or CURB-65/AMBU-65 score 0–2), or a RTI not fulfilling any of these classifications (RTI—Other) (Supplement S2).^11^ Guideline adherence and prolonged treatment for RTI—Other were assessed based on the CAP-m treatment guidelines. Patients with a SSTI were classified as having either a panaritium/paronychia or cellulitis/erysipelas (Supplement S2).^12,13^

### Study outcomes

The primary outcome was prolonged antibiotic treatment. This was calculated separately for UTI, RTI, and SSTI and based on nationally available guidelines (Supplement S3, Table 2).^9–13^ To calculate the LOT, the duration was measured from the initiation of antibiotic treatment at hospitalization until the end of a consecutive period of antibiotic prescriptions, including discharge antibiotics prescribed in the hospitals’ electronic prescribing system. When stop dates were missing from in-hospital antibiotics, the discharge date was used. Preoperative antibiotic prophylaxis prescriptions were excluded, defined as cefazolin prescriptions for ≤24 hours or prescribed by anesthesiologists or surgeons around surgical procedures. Consecutive antibiotic treatment was defined as antibiotic prescriptions with a gap of ≤24 hours between the stop and start date of a new prescription. Guideline adherence was defined as treatment durations within 0.5 days below the minimum to 0.5 days above the upper recommended range. To determine prolonged treatment, the upper limit of the guideline-recommended duration was used, with an additional margin of 0.5 days added to correct for administration time (Table 2).

**Table 1.** Baseline characteristics

Diagnosis Urinary tract infection	No prolonged treatment N = 3,354	Prolonged treatment N = 561	Overall N = 3,915	*P*-value
**Sex**				<.001
Female	1,868 (55.7%)	218 (38.9%)	2,086 (53.3%)	
Male	1,486 (44.3%)	343 (61.1%)	1,829 (46.7%)	
**Age** Median [Q1, Q3]	77 [66;84]	76 [68;84]	77 [66;84]	.383
**Body mass index (BMI)** (kg/m^2^) Median [Q1, Q3]	25.7 [22.8;29.3]	25.9 [22.9;29.2]	25.7 [22.9;29.3]	.983
Missing	646 (19.3%)	66 (11.8%)	712 (18.2%)	
Pregnant	63 (1.9%)	6 (1.1%)	69 (1.8%)	.240
Treatment restriction	1,546 (46.1%)	267 (47.6%)	1,813 (46.3%)	.540
**History of**				
COPD	306 (9.1%)	65 (11.6%)	371 (9.5%)	.077
Diabetes mellitus	952 (28.4%)	137 (24.4%)	1,089 (27.8%)	.059
Heart failure	397 (11.8%)	92 (16.4%)	489 (12.5%)	.003
HIV infection	8 (0.2%)	1 (0.2%)	9 (0.2%)	1.000
Kidney, liver, lung, or heart transplantation	64 (1.9%)	14 (2.5%)	78 (2.0%)	.448
Liver cirrhosis	54 (1.6%)	8 (1.5%)	62 (1.6%)	.888
Renal failure (eGFR<30 ml/min)	578 (17.2%)	120 (21.4%)	698 (17.8%)	.020
**Use of immunosuppressive medication**	352 (10.5%)	80 (14.3%)	432 (11.0%)	.010
**C-reactive protein (CRP) at first measurement** (mg/L) Median [Q1, Q3]	84 [23;170]	93 [37.5;176]	85 [25;170]	.011
Missing	126 (3.8%)	22 (3.9%)	148 (3.8%)	
**Leukocytes at first measurement** (10 ^ 9/L) Median [Q1, Q3]	11.7 [8.5;15.8]	12.5 [9.2;16.6]	11.8 [8.6;15.9]	.002
Missing	75 (2.2%)	13 (2.3%)	88 (2.2 %)	
**Early Warning Score (EWS) during the first 24 hours of hospitalization**	2 [1;4]	2 [1;4]	2 [1;4]	.185
Missing	252 (7.5%)	42 (7.5%)	294 (7.5%)	
**Fever during the first 24 hours of hospitalization**	1,965 (58.6%)	264 (47.1%)	2,229 (56.9%)	<.001
Missing	70 (2.1%)	23 (4.1%)	93 (2.4%)	
**Systemic antibiotic use prior to admission**	1,417 (42.2%)	241 (43.0%)	1,658 (42.4%)	.788

Diagnosis Respiratory tract infection	No prolonged treatment N = 2,240	Prolonged treatment N = 2,203	Overall N = 4,443	*P*-value
**Sex**				.069
Female	1,001 (44.7%)	924 (41.9%)	1,925 (43.3%)	
Male	1,239 (55.4%)	1,279 (58.1%)	2,518 (56.7%)	
**Age** Median [Q1, Q3]	75 [65;83.3]	74 [64;82]	74 [64.5;83]	<.001
**BMI** (kg/m^2^) Median [Q1, Q3]	25.0 [22.0;28.8]	24.9 [22.2;28.5]	25.0 [22.0;28.7]	.814
Missing	471 (21.0%)	420 (19.1%)	891 (20.1%)	
**Pregnant**	6 (0.3%)	5 (0.2%)	11 (0.2%)	1.000
**Treatment restriction**	1,216 (54.3%)	1,107 (50.2%)	2,323 (52.3%)	.008
**History of**				
COPD	658 (29.4%)	559 (25.4%)	1,217 (27.4%)	.003
Diabetes mellitus	530 (23.7%)	474 (21.5%)	1,004 (22.6%)	.094
Heart failure	524 (23.4%)	439 (19.9%)	963 (21.7%)	.006
HIV infection	4 (0.2%)	8 (0.4%)	12 (0.3%)	.262
Kidney, liver, lung, or heart transplantation	14 (0.6%)	17 (0.8%)	31 (0.7%)	.684
Liver cirrhosis	38 (1.7%)	33 (1.5%)	71 (1.6%)	.683
Renal failure (eGFR < 30 ml/min)	272 (12.1%)	244 (11.1%)	516 (11.6%)	.288
**Use of immunosuppressive medication**	234 (10.4%)	228 (10.3%)	462 (10.4%)	.955
**CRP at first measurement** (mg/L) Median [Q1, Q3]	99 [39;185]	129 [56;229]	114 [46;203]	<.001
Missing	80 (3.6%)	46 (2.1%)	126 (2.8%)	
**Leukocytes at first measurement** (10 ^ 9/L) Median [Q1, Q3]	12.3 [9.3;16.4]	12.4 [9.2;16.8]	12.4 [9.2;16.6]	.795
Missing	11 (0.5%)	8 (0.4%)	19 (0.4%)	
**EWS during the first 24 hours of hospitalization**	3 [2;5]	3 [2;5]	3 [2;5]	.022
Missing	258 (11.5%)	290 (13.2%)	548 (12.3%)	
**Fever during the first 24 hours of hospitalizatio**n	1,111 (49.6%)	1,255 (57.0%)	2,366 (53.3%)	<.001
Missing	42 (1.9%)	53 (2.4%)	95 (2.1%)	
**Systemic antibiotic use prior to admission**	700 (31.3%)	756 (34.3%)	1,456 (32.8%)	.032

Diagnosis Skin and soft tissue infection	No prolonged treatment N = 1,321	Prolonged treatment N = 199	Overall N = 1,520	*P*-value
**Sex**				.101
Female	539 (40.8%)	94 (47.2%)	633 (41.6%)	
Male	782 (59.2%)	105 (52.8%)	887 (58.4%)	
**Age** median [Q1, Q3]	67 [53;78]	70 [55;80.5]	67 [53;78]	.082
**BMI** (kg/m^2^) Median [Q1, Q3]	27.8 [24.2;32.9]	29.2 [25.1;35.9]	27.9 [24.4;33.2]	.008
Missing	266 (20.1%)	33 (16.6%)	299 (19.7%)	
**Pregnant**	3 (0.2%)	0 (0%)	3 (0.2%)	1.000
**Treatment restriction**	358 (27.1%)	60 (30.2%)	418 (27.5%)	.416
**History of**				
COPD	78 (5.9%)	24 (12.1%)	102 (6.7%)	.002
Diabetes mellitus	312 (23.6%)	50 (25.1%)	362 (23.8%)	.707
Heart failure	140 (10.6%)	29 (14.6%)	169 (11.1%)	.123
HIV infection	4 (0.3%)	0 (0%)	4 (0.3%)	1.000
Kidney, liver, lung, or heart transplantation	8 (0.6%)	1 (0.5%)	9 (0.6%)	1.000
Liver cirrhosis	18 (1.4%)	3 (1.5%)	21 (1.4%)	.749
Renal failure (eGFR < 30 ml/min)	87 (6.6%)	15 (7.5%)	102 (6.7%)	.728
**Use of immunosuppressive medication**	92 (7.0%)	15 (7.5%)	107 (7.0%)	.884
**CRP at first measurement** (mg/L) Median [Q1, Q3]	96 [37;180]	136 [50;238]	100 [39;190]	<.001
Missing	81 (6.1%)	6 (3.0%)	87 (5.7%)	
**Leukocytes at first measurement** (10 ^ 9/L) Median [Q1, Q3]	11.5 [8.7;15.4]	13.0 [9.6;17.2]	11.7 (8.9;15.6]	.004
Missing	52 (3.9%)	1 (0.5%)	53 (3.5%)	
**EWS during the first 24 hours of hospitalization**	2 [1;3]	2 [1;4]	2 [1;3]	<.001
Missing	109 (8.3%)	22 (11.1%)	131 (8.6%)	
**Fever during the first 24 hours of hospitalization**	567 (42.9%)	112 (56.3%)	679 (44.7%)	.001
Missing	14 (1.1%)	0 (0%)	14 (0.9%)	
**Systemic antibiotic use prior to admission**	756 (57.2%)	112 (56.3%)	868 (57.1%)	.861

**Table 2.** Length of therapy, guideline adherence, and prolonged treatment per infection

Diagnosis group	Total (n)	Length of therapy in days (median, IQR)	Recommended course duration^a^	Guideline adherence (n; %)^b^	Prolonged treatment (n; %)^c^	Prolonged treatment duration in days (median, IQR)^d^
**UTI—Cystitis**	316	4.5 (3.5; 6.0)	5–7 days^g^	53 (16.8)	59 (18.7)	2.2 (1.0;4.8)
**UTI—Systemic symptoms**	3,031	10.1 (6.7;13.4)	7–14 days	2,007 (66.2)	299 (9.9)	1.1 (0.4;2.0)
**UTI—CAD**	263	12.8 (9.1;14.2)	7–14 days	170 (64.6)	48 (18.3)	1.3 (0.6;2.0)
**UTI—Other^e^**	305	6.8 (4.0;10.6)	5–7 days^g^	27 (8.9)	155 (50.8)	4.2 (1.8;6.1)
**RTI—HAP**	196	6.1 (4.4;7.9)	5–7 days	33 (16.8)	53 (27.0)	2.5 (1.4;6.7)
**RTI—Aspiration pneumonia**	488	5.8 (4.4;7.2)	5 days	82 (16.8)	273 (55.9)	1.6 (0.8;3.7)
**RTI—CAP-s**	540	6.0 (4.7;7.3)	5 days	122 (22.6)	309 (57.2)	1.6 (0.9;3.5)
**RTI—CAP-m**	1,560	5.6 (4.6;7.3)	5–7 days^h^	400 (25.6)	762 (48.8)	1.7 (0.8;3.6)
**RTI—Other^f^**	1,659	5.7 (4.4;7.3)	5–7 days^h^	374 (22.5)	806 (48.6)	1.8 (0.9;4.4)
**SSTI—Cellulitis/ erysipelas**	1,364	12.7 (9.5;14.0)	10–14 days	827 (60.6)	187 (13.7)	1.2 (0.5;2.8)
**SSTI—Panaritium/ paronychia**	156	9.1 (7.1;11.4)	7–14 days	113 (72.4)	12 (7.7)	1.5 (0.5;1.9)
**Total**	9,878					

n, number of patients; IQR, interquartile range; UTI, urinary tract infection; UTI-CAD, catheter-associated urinary tract infection; RTI, respiratory tract infection; CAP-m, community-acquired pneumonia mild-to-moderate; CAP-s, community-acquired pneumonia severe; HAP, hospital-acquired pneumonia; PSI, pneumonia severity index; SSTI, skin and soft tissue infection.

a. In accordance with Dutch guidelines^9–13^; b. Guideline adherence was defined as treatment durations within 0.5 days below the minimum to 0.5 days above the upper recommended range; c. Prolonged treatment was defined as the upper limit of the guideline-recommended duration, with an additional 0.5 days margin to correct for administration time; d. Determined only in patients with prolonged treatment. Excess days were calculated as the difference between the length of therapy and the guideline-recommended upper limit plus 0.5 days; e. Other urinary tract infection; f. Other pneumonia or CAP without an PSI, AMBU-65 or CURB-65 score; g. 7 days for women with diabetes mellitus, women receiving immunosuppressive medication, pregnant women not treated with amoxicillin clavulanic acid, or male patients. 5 days for pregnant women receiving amoxicillin clavulanic acid or healthy nonpregnant women; h. 7 days in case of doxycycline use.

To address the secondary objective, identification of risk factors associated with prolonged antibiotic treatment, we used the same outcome of prolonged treatment. The following risk factors were included: age, sex, body mass index, medical specialty (surgical vs nonsurgical), hospital of admission, intravenous-to-oral antibiotic switch (oral-only, intravenous-only, or switch), antibiotic use at admission, being discharged before guideline-recommended treatment duration, positive blood cultures, positive sputum cultures, positive urinary cultures, positive wound cultures, infectious diagnosis category, use of systemic immunosuppressive medication, initial C-reactive protein (CRP) level, change in CRP between the final and prior maximum measurement (increased/remained the same level, decreased, or not measured), initial leukocyte count, change in leukocyte count between the final and prior maximum measurement (increased/remained the same level, decreased, or not measured), presence of fever during the first 24 hours of hospitalization, absence of fever during the last 24 hours measured within the guideline-recommended treatment duration, highest Early Warning Score (EWS) during the first 24 hours of hospitalization, and highest EWS during the last 24 hours measured (see Supplement S2 for definitions).

### Data collection

All four hospitals have ChipSoft HiX as their EHR and prescribing system. Pseudonymized data was extracted from the EHR using CTCue (version v4.15.2). CTcue is a text-mining software application designed to construct rule-based queries and perform contextual analysis for patient selection and data extraction from both structured and unstructured EHR data.^17^ The following parameters were extracted uniformly across all hospitals, using predefined queries developed by one researcher (SvdE): baseline characteristics, including treatment restriction defined as the presence of any documented care limitation such as “do not resuscitate” or “no admission to the intensive care unit,” hospital data, antibiotics prescribed during hospitalization and at discharge, laboratory results, vital signs, microbiology findings, and (co-)infections diagnoses.

### Statistical analysis

Continuous baseline characteristics were reported using mean and standard deviation or median and interquartile range, as appropriate. Categorical baseline characteristics were reported as frequencies and percentages. Differences in characteristics between patients receiving prolonged treatment and those not receiving prolonged treatment were analyzed using the chi-square test, Fisher’s exact test, Student’s t-test, or Mann–Whitney *U*-test, as appropriate. Multiple imputation of missing predictors was performed, with the number of imputation sets determined by the covariate with the highest percentage of missingness. Accordingly, twenty imputation sets were used in the analyses. Backward stepwise multivariable logistic regression was used to identify risk factors for prolonged treatment for RTI, UTI, and SSTI separately. Variable selection was based on Wald tests, and variables with a *P* value ≥ .2 were removed from the model. Model performance was evaluated using the C-statistic and calibration slope (“shrinkage factor”). Internal validity was assessed through 50 bootstrap replications to obtain the shrinkage factor and the bias-corrected estimates of the C-statistic. Based on the bias-corrected C-statistic, the model was classified as poor (<0.6), fair (0.6–0.7), moderate (0.7–0.8), good (0.8–0.9), or strong (0.9–1.0). Effect estimates were reported as odds ratios (ORs) with 95% confidence intervals (CIs), including both unadjusted and adjusted ORs (aOR). The unadjusted ORs were corrected for optimism using the shrinkage factor obtained through bootstrapping, resulting in the aORs.

## Results

### Baseline characteristics

**Figure 1.**
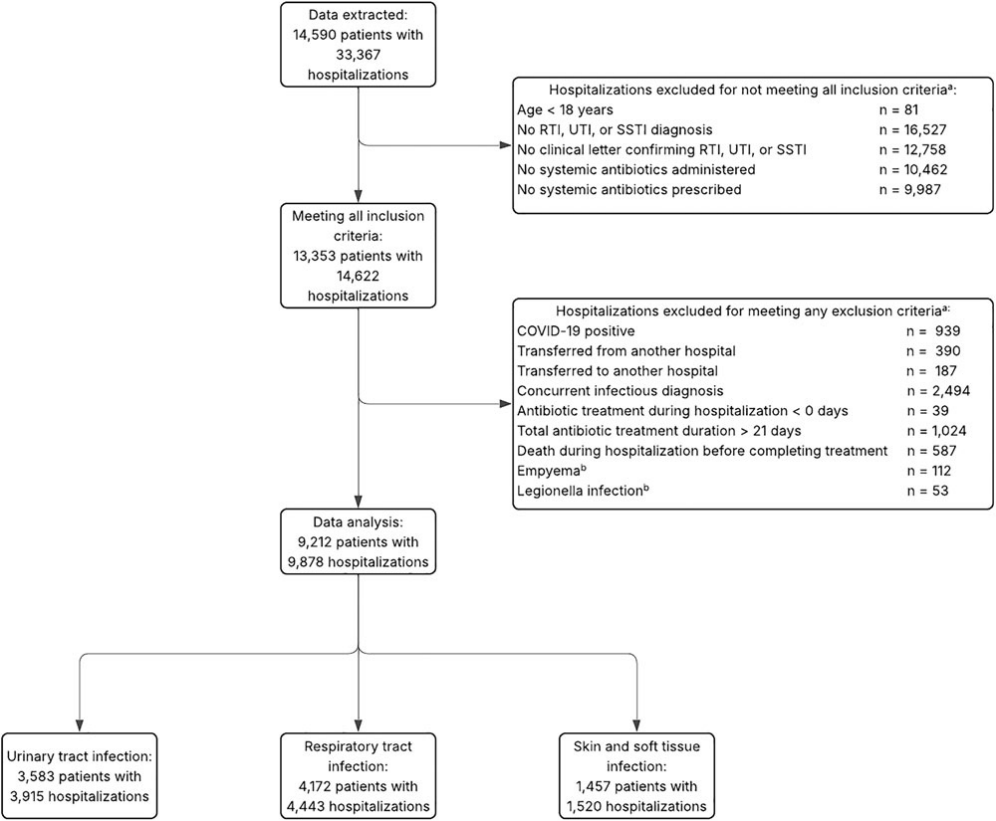
Flowchart. a. Criteria may overlap; b. Exclusion criteria apply only to patients with a respiratory tract infection.

A total of 14,590 patients with 33,367 hospitalizations were extracted. Of these, 23,489 hospitalizations were excluded, including 2,494 due to the presence of a concurrent infectious diagnosis during hospitalization (Figure 1). Overall, 9,212 patients with 9,878 hospitalizations were included, among whom 39.6% were hospitalized with a UTI, 44.9% with a RTI, and 15.4% with a SSTI (Figure 1). In 30.0% of hospitalizations, the duration of antibiotic treatment exceeded the recommendations (Table 1). Among UTI patients receiving prolonged treatment, 61.1% were male, compared to 44.3% of those not receiving prolonged treatment. For RTIs and SSTIs, 58.1% and 52.8% of patients receiving prolonged treatment were male, compared to 55.4% and 59.2% among those not treated for longer than the recommended duration. Patients with prolonged treatment had higher baseline CRP levels and a higher proportion presented with fever within the first 24 hours of hospitalization (Table 1).

### Guideline adherence and prolonged treatment duration

Guideline adherence ranged from 8.9% for UTI—Other to 72.4% for panaritium/paronychia, while prolonged treatment varied between 7.7% for panaritium/paronychia and 57.2% for CAP-s (Table 2). Doxycycline use was 9.7% in CAP-m and 9.6% in RTI—Other. Prolonged antibiotic treatment was particularly common among RTI patients (49.6%, Table 2) and accounted for 31.0% of total antibiotic days, compared with 7.4% for UTIs and 3.3% for SSTIs. Overall, a trend toward shorter median treatment durations for both CAP-m and CAP-s was observed in 2023 compared to 2021 (Supplement S4). Among patients with prolonged treatment, 45.3% to 100% were prescribed antibiotics at discharge (Table 3), compared with 36.1% to 98.2% among those with guideline-adherent durations (Supplement S5). Applying guideline recommendations for “UTI with systemic symptoms” instead of “cystitis” to patients categorized as UTI-Other increased guideline adherence from 8.9% (n = 27) to 53.1% (n = 162) and reduced prolonged treatment from 50.8% (n = 89) to 3.6% (n = 11).

**Table 3.** Patients with prolonged treatment and postdischarge treatment

Diagnosis group	Total (n)	Treatment courses with postdischarge antibiotics (n; %)	Length of postdischarge therapy in days (median, IQR)
**UTI—Cystitis**	59	47 (79.7)	6.2 (3.5; 8.1)
**UTI—Systemic symptoms**	299	254 (84.9)	10.4 (7.6;12.3)
**UTI—CAD**	48	42 (87.5)	9.4 (7.0;11.3)
**UTI—Other^a^**	155	126 (81.3)	7.5 (5.4;9.6)
**RTI—HAP**	53	24 (45.3)	4.8 (2.2;6.6)
**RTI—Aspiration pneumonia**	273	124 (45.4)	3.5 (2.3;5.4)
**RTI—CAP-s**	309	158 (51.1)	3.4 (2.3;5.3)
**RTI—CAP-m**	762	549 (72.0)	4.3 (2.5;5.5)
**RTI—Other^b^**	806	498 (61.8)	4.4 (2.5;6.3)
**SSTI—Cellulitis/erysipelas**	187	165 (88.2)	8.4 (6.4;11.0)
**SSTI—Panaritium/paronychia**	12	12 (100)	12.5 (10.5;13.5)

n, number of patients, IQR, interquartile range; UTI, urinary tract infection; UTI-CAD, catheter-associated urinary tract infection; RTI, respiratory tract infection; CAP-m, community-acquired pneumonia mild-to-moderate severe; CAP-s, community-acquired pneumonia severe; HAP, hospital-acquired pneumonia; PSI, pneumonia severity index; SSTI, skin and soft tissue infection.

a. Other urinary tract infection; b. Other pneumonia or CAP without an PSI, AMBU-65 or CURB-65 score.

### Risk factors

**Figure 2.**
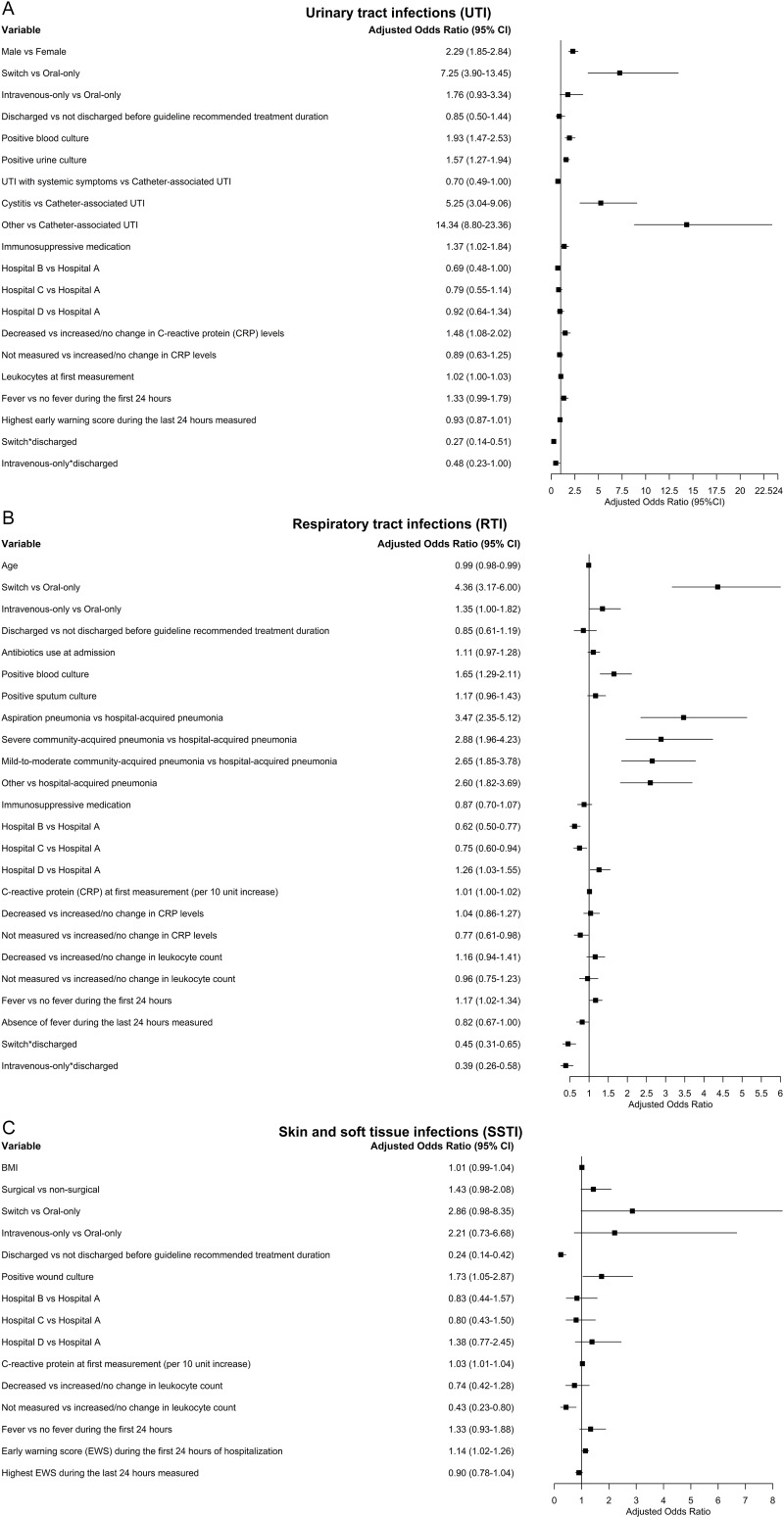
Risk factors associated with prolonged treatment, A: urinary tract infections, B: respiratory tract infections, and C: skin and soft tissue infections.

Figure 2 presents the aORs, calculated using the shrinkage factor (0.99 for UTI, 0.99 for RTI, and 0.95 for SSTI), for potential risk factors associated with prolonged treatment. For the ORs not adjusted by the shrinkage factor, see Supplement S6, and for the full initial prediction model prior to stepwise selection, see Supplement S7.

Among patients with either UTIs or RTIs, an intravenous-to-oral switch and a positive blood culture were significantly associated with higher odds of prolonged treatment, while discharge prior to completing the guideline-recommended treatment duration following an intravenous-to-oral switch was associated with lower odds (Figure 2a, 2b). For UTIs, male sex, a positive urine culture, and a cystitis or UTI—Other (compared to UTI—Catheter) resulted in higher odds of prolonged treatment. For RTIs, fever within the first 24 hours of hospitalization, admission to hospital D (compared to hospital A), and a diagnosis other than HAP were associated with receiving prolonged antibiotic treatment. The optimism-corrected C-statistics for the models were 0.78 (95% CI: 0.76–0.80) for UTIs and 0.71 (95%CI: 0.70–0.73) for RTIs, indicating moderate discrimination. For patients with SSTIs, factors associated with increased odds included a positive wound culture and a higher EWS in the first 24 hours (Figure 2c). In contrast, discharge prior to the guideline-recommended duration resulted in a lower odd of prolonged treatment. This model yielded an optimism-corrected C-statistic of 0.68 (95% CI: 0.64–0.72), indicating fair discrimination.

## Discussion

Prolonged antibiotic treatment durations were observed in 49.6% of patients hospitalized with a RTI, compared to 14.3% for UTIs and 13.1% for SSTIs. The highest proportion of patients receiving prolonged antibiotic treatment were those diagnosed with aspiration pneumonia, severe CAP or mild-to-moderate CAP. Additionally, a high proportion of patients classified as UTI—Other received prolonged antibiotic treatment. Risk factors for prolonged treatment included positive cultures, intravenous-to-oral switch, male sex (in UTI patients), and diagnoses of aspiration pneumonia or CAP among RTI patients, as well as cystitis among UTI patients. An intravenous-to-oral switch, positive cultures, high proportion of males among UTI patients receiving prolonged antibiotic treatment, and variations in guideline concordance across the infectious diagnoses represent potential targets for ASPs.

Consistent with previous studies, RTI patients frequently receive antibiotic therapy that exceeds the recommended duration.^4,18,19^ In our study, 51.0% of CAP patients received antibiotic therapy that exceeded the recommended duration of five to seven days. Prolonged antibiotic use is not without consequence, as each additional day of treatment has previously been associated with 4% increased odds of experiencing an adverse drug event, resulting in a substantial proportion of patients at increased risk.^20^ These adverse events, including gastrointestinal and dermatological symptoms, emphasize the clinical importance of preventing inappropriate prolonged antibiotic therapy.^20^ Additionally, the frequent prescription of antibiotics at discharge is consistent with our findings, in which 60.1% of CAP patients received antibiotics following hospital discharge.^19^ This highlights the importance of including discharge prescriptions when calculating the total duration of treatment.

An intravenous-to-oral switch was identified as a risk factor for prolonged treatment. This suggests that prescribers may not consistently include the days of intravenous therapy when calculating the total length of antibiotic treatment, possibly due to challenges in determining the exact start date of antibiotic treatment. A primary focus of ASPs is to support the appropriate switching from intravenous to oral antibiotic therapy.^8^ Future antibiotic stewardship strategies should not only promote timely switching but also ensure that the cumulative duration of both intravenous and oral therapy is consistently considered. This is particularly important for patients who remain hospitalized longer than the guideline-recommended duration of therapy. In cases of RTIs and UTIs, discharge before completing the guideline-recommended treatment duration following an intravenous-to-oral switch was associated with lower odds of prolonged antibiotic treatment. This suggests that discharge represents an additional opportunity to reassess and optimize antibiotic therapy, a potential that has been previously recognized.^1,21^

Male UTI patients had higher odds of receiving prolonged antibiotic therapy compared to female patients. For patients with a UTI with systemic symptoms, 203 out of 299 patients with prolonged antibiotic treatment were males. This may be partly explained by Dutch guideline recommendations, which recommend a 7–14-day course for women, depending on the selected antibiotic, whereas men are recommended a 14-day course.^9^ Consequently, in case of concerns about the treatment recommendation, men may be more likely to receive treatment exceeding fourteen days. This could be the case if total treatment duration is not properly considered following an intravenous-to-oral switch or after de-escalation.

A strength of our study is the inclusion of data from four secondary hospitals in the Netherlands, which enhances the generalizability of our findings. Additionally, the identified risk factors could be useful to optimize ASP and support efforts to reduce unnecessary prolonged antibiotic therapy. In particular, ASPs could focus on improving treatment duration for RTIs. Systematic data extraction of antibiotic treatment duration and indication of antibiotic treatment is currently not practiced in the Netherlands. The data collection and analysis approach used in this study could be a valuable tool for monitoring prescribing practices and determining the impact of ASPs. The individual results have been shared with the participating hospitals, where they can be used to improve local prescribing practices. Moreover, the inclusion of discharge prescriptions in the calculation of the LOT increased the completeness of the results.

Our study has several limitations. First, the participating hospitals did not implement mandatory indication registration for antibiotic prescriptions, increasing the risk of misclassification due to reliance on free-text entries and contributing to a large proportion of patients being categorized as “other.” To reduce the probability of incorrect inclusions or wrong labeling of infections in the study, ICD-10 codes were used together with free-text entries to improve the reliability of the infectious diagnosis. However, this approach may have resulted in underrepresentation of certain infection types. Second, exclusion of patients with concurrent infectious diagnoses may limit the generalizability of the findings. However, this has likely resulted in a study population that more accurately represents the infections of interest. In patients with multiple infections without indication registration, it was not feasible to determine which antibiotic was prescribed for each infection. Third, treatment duration was based on documented prescription start and stop dates, which could have underestimated total treatment duration and prolonged treatment. This could occur when the final antibiotic was prescribed as a once-daily dose and the recorded stop date coincided with the time of administration. Fourth, reasons for non-guideline-concordant treatment durations were not systematically documented and, therefore, could not be assessed. Some patients classified as having received prolonged antibiotic treatment could have had valid clinical indications. For example, if empirical treatment failed to cover the identified pathogen, or if there was a lack of clinical improvement. It remains uncertain to what extent such deviations were appropriate.

To conclude, there was a high period prevalence of patients hospitalized with community-onset RTIs who received prolonged antibiotic treatment durations. Prolonged treatment was independently associated with the intravenous-to-oral antibiotic switch, positive cultures, and infectious diagnosis of the patient. These findings highlight the need for strategies to optimize antibiotic treatment durations, and the identified risk factors should be considered in ASPs. Future research should focus on studying the barriers and facilitators influencing antibiotic treatment duration, as well as assessing the appropriateness of non-guideline concordance and its impact on prolonged antibiotic treatment.

## Supporting information

10.1017/ash.2026.10330.sm001 van den Eijnde et al. supplementary material van den Eijnde et al. supplementary material

## Supplementary material

The supplementary material for this article can be found at https://doi.org/10.1017/ash.2026.10330.

## Research transparency and reproducibility

The data set is available from the corresponding author on reasonable request.
