# Supplementary material for: Prolonged antibiotic treatment durations for community-onset infections in Dutch hospitals
Source: Antimicrob Steward Healthc Epidemiol. 2026 Mar 27;6(1):e70. doi: 10.1017/ash.2026.10330 (PMC13104520; doi:10.1017/ash.2026.10330)
Supplement: van den Eijnde et al. supplementary material [file S2732494X26103301sup001.docx]

Supplement to:

Prolonged antibiotic treatment durations for community-onset infections in Dutch hospitals

**Table of contents**

[S1. ICD-10 codes of infectious diagnoses 2](#_Toc220837050)

[S2. Definitions of variables 3](#_Toc220837051)

[S3. Guideline recommendations 7](#_Toc220837052)

[S4. Median antibiotic treatment durations from 2021 to 2023 8](#_Toc220837053)

[S5. Patients with treatment durations in concordance with guidelines and post-discharge treatment 10](#_Toc220837054)

[S6. Risk factors associated with prolonged treatment 11](#_Toc220837055)

[S7. Full prediction model 14](#_Toc220837056)

[References 17](#_Toc220837057)

# S1. ICD-10 codes of infectious diagnoses

Table S1. ICD-10 codes of infectious diagnoses

| Infection diagnosis | ICD-10 codes starting with^1^ |
| --- | --- |
| Bone and joint infection | M01, T84.5, M86, T84.7 |
| Cardiovascular infection | T82.7, J98.5, I72.9, I31.9, I30, T82.6, I38, I39.8, I33 |
| Gastro-intestinal infection | A09, A04 |
| Genital infection | N71, N73, A64, N76 |
| Intra-abdominal infection | K83, K81, K65, K75, K35 |
| Meningitis / encephalitis | G04, G06, G00, G03 |
| Neutropenic fever | D70 |
| Respiratory tract infection* | J18, J15, J69 |
| Respiratory tract infection, other | J85, J86 |
| Skin and soft tissue infection* | L03, A46 |
| Skin and soft tissue infection, other | M72.6, T81.4, T79.3 |
| Urinary tract infection* | N39.0, N30.0, N30.8, N30.9, N10, O86.2, O23.1, O23.4, T83.5 |
| Urinary tract infection, other | N41 |

*Used to include patients with a respiratory tract infection, skin and soft tissue infection or urinary tract infection

# S2. Definitions of variables

Table S2. Definitions of variables

| Category | Variables | Definitions* |
| --- | --- | --- |
| Inclusion criteria | COVID-19 positive | Confirmed COVID-19 by positive test within 48 hours before or after hospital admission and recorded in microbiology data  OR  Clinical diagnosis of COVID-19 with start date 48 hours before or after hospital admission |
| Diagnosis category | UTI with a urinary catheter | 1. ICD-10 diagnosis T83.5 (Infection and inflammatory reaction due to prosthetic device, implant and graft in urinary system) OR  2. Documentation of urinary (intermittent) catheter use prior to admission in discharge letter |
|  | UTI with systemic symptoms | 1. ICD-10 diagnosis N10 (Acute tubulo-interstitial nephritis) OR  2. Discharge letter confirming pyelonephritis OR  3. Discharge letter confirming urinary tract sepsis OR  4. Discharge letter confirming complicated urinary tract infection OR  5. Documented fever (≥38.0°C) in vital signs during hospitalization OR  6. In-hospital consultation note confirming delirium OR  7. In-hospital consultation note confirming flank pain |
|  | Cystitis | 1. ICD-10 diagnosis N30.0 (Acute cystitis), N30.8 (Other cystitis), or N30.9 (Cystitis, unspecified) OR  2. Discharge letter confirming cystitis OR  3. Prescription of nitrofurantoin or fosfomycin during first 48 hours of hospitalization |
|  | UTI – Other | Not meeting the criteria for classification as catheter-associated UTI, UTI with systemic symptoms, or cystitis |
|  | RTI – Hospital acquired pneumonia | Discharge letter confirming hospital acquired pneumonia |
|  | RTI – Aspiration pneumonia | 1. Discharge letter confirming aspiration pneumonia OR  2. ICD-10 diagnosis J69 (Pneumonitis due to solids and liquids) |
|  | RTI – CAP-s | Discharge letter or consultation note confirming Pneumonia Severity Index (PSI) class 5, or CURB-65/AMBU-65 score of 3-5 OR  Discharge letter confirming pneumosepsis |
|  | RTI – CAP-m | Discharge letter or consultation note confirming PSI class 1-4 or CURB-65/AMBU-65 score 0-2 |
|  | RTI – Other | Not meeting the criteria for classification as hospital acquired pneumonia, aspiration pneumonia, CAP-s, or CAP-m |
|  | SSTI – Panaritium/paronychia | 1. ICD-10 diagnosis L03.0 (Cellulitis of finger and toe) OR  2. Discharge letter confirming panaritium or paronychia |
|  | SSTI – Cellulitis / erysipelas | 1. ICD-10 diagnosis L03.1 (Cellulitis of other parts of limb), L03.2 (Cellulitis of face), L03.3 (Cellulitis of trunk), L03.8 (Cellulitis of other sites), or L03.9 (Cellulitis, unspecified) OR  2. Discharge letter confirming cellulitis / erysipelas |
| Determinants | Age | Age at admission |
|  | Sex | Sex (female or male) |
|  | Body mass index (BMI) (kg/m^2^) | 1. BMI recorded during hospitalization OR  2. BMI recorded within 7 days prior to admission OR  3. BMI recorded within 1 month prior to admission OR  4. BMI recorded within 1 year prior to admission |
|  | Medical specialty | Medical specialty confirming the infection diagnosis in the discharge letter |
|  | Hospital of admission | Hospital of admission |
|  | Intravenous-to-oral switch | Oral-only, intravenous-only, or a switch from intravenous to oral antibiotic therapy during hospitalization or at discharge |
|  | Antibiotic use at admission | Systemic antibiotic use at admission documented in the electronic health record as ‘home medication’, ‘verified medication at admission’, OR ‘requested medication via National Exchange Point (Landelijk Schakelpunt (LSP))’ |
|  | Being discharged before guideline recommended treatment duration | Discharge occurring prior to guideline-recommended treatment duration, with recommendations varying by the infection diagnosis of the patient |
|  | Positive blood cultures | Positive blood culture indicating bacterial infection, as recorded in microbiology data |
|  | Positive sputum cultures | Positive sputum culture indicating bacterial infection, as recorded in microbiology data |
|  | Positive urinary cultures | Positive urinary culture indicating bacterial infection, as recorded in microbiology data |
|  | Positive wound cultures | Positive wound culture indicating bacterial infection, as recorded in microbiology data |
|  | Diagnosis | See Diagnosis category |
|  | Immunosuppressive medication | Prescription of systemic Anatomical Therapeutic Chemical (ATC) codes L01, L02, L03, L04 |
|  | Initial C-reactive protein (CRP) level | First CRP measurement recorded during hospitalization |
|  | Change in CRP | Difference between the final CRP measurement within the guideline-recommended duration and the prior maximum measurement, categorized as increased/remained the same, decreased, or not measured |
|  | Initial leukocyte count | First leukocyte count recorded during hospitalization |
|  | Change in leukocyte count | Difference between the final leukocyte count within the guideline-recommended duration and the prior maximum measurement, categorized as increased/remained the same, decreased or not measured |
|  | Presence of fever during the first 24 hours of hospitalization | Fever (≥38.0°C) recorded within the first 24 hours of hospitalization |
|  | Absence of fever during the last 24 hours measured | No fever (<38.0°C) recorded during the last 24 hours measured within the guideline-recommended duration |
|  | Highest Early Warning Score (EWS) during the first 24 hours of hospitalization | Maximum EWS during the first 24 hours of hospitalization |
|  | Highest EWS during the last 24 hours measured | Maximum EWS during the last 24 hours measured within guideline-recommended duration |

*Definitions include synonyms and terms that cannot appear together in the same result. Example for pyelonephritis:

1. The content must include one of the following terms: pyelonefritis, pyelonephritis, pyelo nefritis

2. The content must not include phrases such as: “no indications of pyelonephritis", "no suspicion of pyelonephritis", “in medical history, pyelonephritis”

# S3. Guideline recommendations

| Diagnosis group | Summary Dutch guideline recommendations |
| --- | --- |
| UTI – Cystitis^2,3^ | - Single-dose therapy for women treated with fosfomycin^*^ - Three days for women treated with trimethoprim^*^ - Five days for pregnant women treated with amoxicillin–clavulanic acid and healthy non-pregnant women - Seven days for women with diabetes mellitus, women receiving immunosuppressive therapy, pregnant women not treated with amoxicillin–clavulanic acid, and male patients. |
| UTI – Systemic symptoms^3,4^ | - Seven days for women treated with ciprofloxacin - Ten to fourteen days for women treated with co-trimoxazole or a beta-lactam. Seven days of treatment may be considered if the patient is hemodynamically stable and afebrile for at least 48 hours. - Fourteen days for men. Seven days of treatment may be considered if the patient is hemodynamically stable and afebrile for at least 48 hours.   Switch to oral therapy when clinically stable. |
| UTI – CAD^3,4^ | Follow the same recommendations as for UTI with systemic symptoms |
| RTI – HAP^3,5^ | Five to seven days. Switch to oral therapy after 48 – 72 hours when clinically stable. |
| RTI – Aspiration pneumonia^3,6^ | Five days in patients with a good clinical response. Switch to oral therapy when clinically improved. |
| RTI – CAP-s^3,6^ | Five days in patients with a good clinical response. Switch to oral therapy when clinically improved. |
| RTI – CAP-m^3,6^ | Five days for patients with good clinical response. A maximum of seven days for patients treated with doxycycline. In case of intravenous therapy, switch to oral therapy when clinically improved. |
| SSTI – Cellulitis/erysipelas^3,7,8^ | Ten to fourteen days. In case of intravenous therapy, switch to oral therapy after one to two days when clinically improved. |
| SSIT – Panaritium/paronychia^3,7^ | Seven to fourteen days. |

*Five days was defined as the recommended treatment duration for the analysis.

# S4. Median antibiotic treatment durations from 2021 to 2023


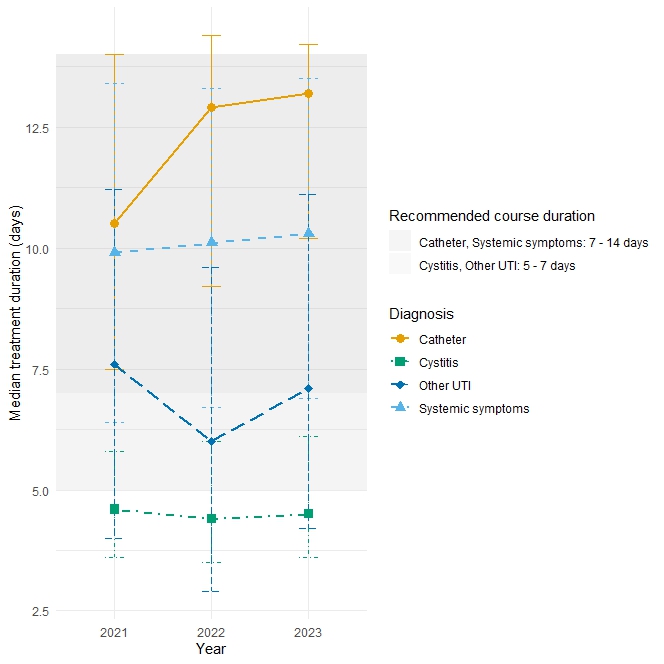


Figure S4a. Median duration of antibiotic treatment for urinary tract infections in 2021-2023


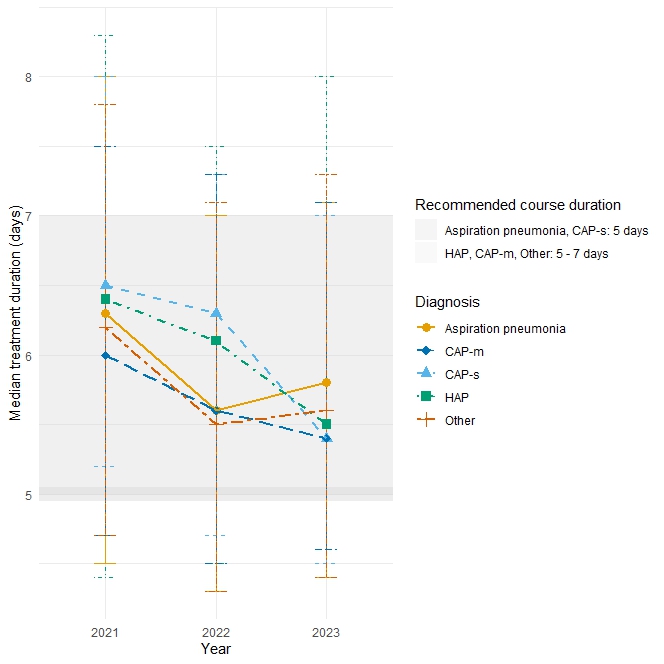


Figure S4b. Median duration of antibiotic treatment for respiratory tract infections in 2021-2023


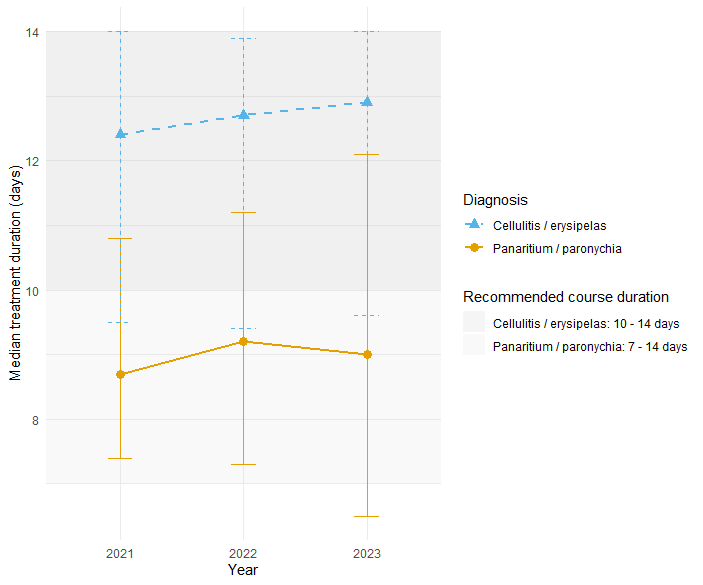


Figure S4c. Median duration of antibiotic treatment for skin and soft tissue infections in 2021-2023

# S5. Patients with treatment durations in concordance with guidelines and post-discharge treatment

Table S5. Patients with treatment durations in concordance with guidelines and post-discharge treatment

| Diagnosis group | Total (n) | Treatment courses with post-discharge antibiotics (n; %) | | Length of post-discharge therapy in days (median, IQR) | | |
| --- | --- | --- | --- | --- | --- | --- |
| UTI – Cystitis | 53​ | | 19 (70.4)​ | | 3.5 (2.9;4.4)​ |  |
| UTI – Systemic symptoms | 2,007​ | | 1,723 (85.8)​ | | 7.3 (5.4;9.4)​ |  |
| UTI – CAD | 170​ | | 143 (84.1)​ | | 7.4 (5.4;9.3)​ |  |
| UTI – Other^a^ | 27​ | | 19 (70.4)​ | | 3.5 (2.9;4.4)​ |  |
| RTI – HAP | 33​ | | 19 (57.6)​ | | 3.5 (1.5;4.5)​ |  |
| RTI – Aspiration pneumonia | 82​ | | 32 (39.0)​ | | 1.8 (1.2;3.4)​ |  |
| RTI – CAP-s | 122​ | | 44 (36.1)​ | | 2.3 (1.4;2.4)​ |  |
| RTI – CAP-m | 400​ | | 251 (62.7)​ | | 2.5 (1.5;3.4)​ |  |
| RTI – Other^b^ | 374 | | 164 (43.9)​ | | 2.4 (1.4;3.5)​ |  |
| SSTI – Cellulitis/erysipelas | 827​ | | 774 (93.6)​ | | 8.5 (6.5;103)​ |  |
| SSTI - Panaritium/paronychia | 113​ | | 111 (98.2)​ | | 6.5 (5.5;8.4)​ |  |

n; number of patients, IQR, interquartile range; UTI, urinary tract infection; UTI-CAD, catheter-associated urinary tract infection; RTI; respiratory tract infection; CAP-m, community-acquired pneumonia mild-to-moderate severe; CAP-s, community-acquired pneumonia severe; HAP, hospital-acquired pneumonia; PSI, pneumonia severity index; SSTI, skin and soft tissue infection.

a. Other urinary tract infection; b. Other pneumonia or CAP without an PSI, AMBU-65 or CURB-65 score.

# S6. Risk factors associated with prolonged treatment

Table S6. Risk factors associated with prolonged treatment (calculated without usage of the shrinkage factor)

| Variables | OR (95% CI)  Urinary tract infection | OR (95% CI)  Respiratory tract infection | OR (95% CI)  Skin and soft tissue infection |
| --- | --- | --- | --- |
| Sex  Female  Male | Ref  2.31 (1.87 – 2.87) | NA | NA |
| Age | NA | 0.99 (0.98 – 0.99) | NA |
| BMI | NA | NA | 1.02 (1.00 – 1.04) |
| Medical specialty discharge letter  Non-surgical  Surgical | NA | NA | Ref  1.46 (1.01 – 2.13) |
| Intravenous-to-oral antibiotic switch  Oral-only  Intravenous-only  Switch | Ref  1.77 (0.93 – 3.37)  7.42 (4.00 – 13.76) | Ref  1.35 (1.01 – 1.82)  4.44 (3.23 – 6.11) | Ref  2.32 (0.77 – 6.99)  3.04 (1.04 – 8.87) |
| Discharged before guideline recommended treatment duration | 0.84 (0.50 – 1.43) | 0.85 (0.61 – 1.18) | 0.22 (0.13 – 0.39) |
| Intravenous-to-oral antibiotic switch * Discharged before guideline recommended treatment duration  Oral-only * Not discharged  Intravenous-only * discharged  Switch * discharged | Ref  0.48 (0.23 – 1.00)  0.26 (0.14 – 0.50) | Ref  0.38 (0.26 – 0.57)  0.44 (0.30 – 0.65) | NA |
| Immunosuppressive medication | 1.37 (1.02 – 1.85) | 0.87 (0.70 – 1.07) | NA |
| C-reactive protein (CRP) at first measurement (per 10 unit increase) | NA | 1.01 (1.00 – 1.02) | 1.03 (1.01 – 1.04) |
| Change in CRP levels  Increased/no change  Decreased  Not measured | Ref  1.48 (1.08 – 2.03)  0.89 (0.63 – 1.24) | Ref  1.04 (0.86 – 1.27)  0.77 (0.61 – 0.97) | NA |
| Leukocytes at first measurement | 1.02 (1.00 – 1.03) | NA | NA |
| Change in leukocyte count  Increased/no change  Decreased  Not measured | NA | Ref  1.16 (0.95 – 1.42)  0.96 (0.75 – 1.23) | Ref  0.72 (0.42 – 1.26)  0.41 (0.22 – 0.76) |
| Early warning score (EWS) during the first 24 hours of hospitalization | NA | NA | 1.15 (1.03 – 1.27) |
| Highest EWS during the last 24 hours measured | 0.93 (0.86 – 1.01) | NA | 1.15 (1.03 – 1.27) |
| Fever during the first 24 hours of hospitalization | 1.34 (1.00 – 1.79) | 1.17 (1.02 – 1.34) | 1.35 (0.95 – 1.91) |
| Absence of fever during the last 24 hours measured | NA | 0.82 (0.67 – 0.99) | NA |
| Antibiotics use at admission | NA | 1.12 (0.97 – 1.28) | NA |
| Positive blood culture | 1.94 (1.48 – 2.55) | 1.66 (1.30 – 2.12) | NA |
| Positive urinary culture | 1.58 (1.28 – 1.95) | NA | NA |
| Positive sputum culture | NA | 1.18 (0.96 – 1.43) | NA |
| Positive wound culture | NA | NA | 1.79 (1.08 – 2.96) |
| Diagnosis  UTI- Catheter  UTI - Systemic symptoms  UTI - Cystitis  UTI - Other | Ref  0.69 (0.48 – 1.00)  5.35 (3.10 – 9.23)  14.79 (9.08 – 24.08) | NA | NA |
| Diagnosis  RTI - HAP  RTI – Aspiration pneumonia  RTI – CAP-s  RTI – CAP-m  RTI - Other | NA | Ref  3.53 (2.39 – 5.21)  2.92 (1.99 – 4.29)  2.68 (1.88 – 3.82)  2.63 (1.85 – 3.74) | NA |
| Hospital  Hospital A  Hospital B  Hospital C  Hospital D | Ref  0.69 (0.48 – 0.99)  0.79 (0.55 – 1.13)  0.92 (0.64 – 1.34) | Ref  0.62 (0.50 – 0.77)  0.75 (0.60 – 0.94)  1.27 (1.03 – 1.56) | Ref  0.82 (0.43 – 1.56)  0.79 (0.43 – 1.48)  1.40 (0.79 – 2.49) |

UTI, urinary tract infection; UTI-CAD, catheter-associated urinary tract infection; RTI; respiratory tract infection; CAP-m, community-acquired pneumonia mild-to-moderate; CAP-s, community-acquired pneumonia severe; HAP, hospital-acquired pneumonia; PSI, pneumonia severity index; SSTI, skin and soft tissue infection; OR: odds ratio; CI: confidence interval; NA: not applicable

# S7. Full prediction model

Table S7. Separate full prediction models for urinary tract infections, respiratory tract infections, and skin and soft tissue infections

| Variables | OR (95% CI)  Urinary tract infection | OR (95% CI)  Respiratory tract infection | OR (95% CI)  Skin and soft tissue infection |
| --- | --- | --- | --- |
| Sex  Female  Male | Ref  2.40 (1.92 – 2.99) | Ref  1.09 (0.95 – 1.24) | Ref  0.86 (0.62 – 1.19) |
| Age | 0.96 (0.89 – 1.03) | 0.99 (0.98 – 0.99) | 1.05 (0.95 – 1.17) |
| Body mass index (BMI) | 0.99 (0.97 – 1.01) | 1.00 (0.99 – 1.01) | 1.01 (0.99 – 1.04) |
| Medical specialty discharge letter  Non-surgical  Surgical | Ref  0.91 (0.72 – 1.15) | Ref  0.95 (0.70 – 1.29) | Ref  1.60 (1.07 – 2.40) |
| Intravenous-to-oral antibiotic switch  Oral-only  Intravenous-only  Switch | Ref  1.77 (0.93 – 3.38)  7.33 (3.94 – 13.64) | Ref  1.35 (1.00 – 1.83)  4.40 (3.19 – 6.05) | Ref  3.26 (0.32 – 32.85)  3.14 (0.30 – 32.47) |
| Discharged before guideline recommended treatment duration | 0.83 (0.49 – 1.41) | 0.85 (0.61 – 1.18) | 0.30 (0.02 – 3.67) |
| Intravenous-to-oral antibiotic switch * Discharged before guideline recommended treatment duration  Oral-only * Not discharged  Intravenous-only * discharged  Switch * discharged | Ref  0.47 (0.23 – 0.99)  0.26 (0.13 – 0.50) | Ref  0.44 (0.30 – 0.65)  0.38 (0.26 – 0.57) | Ref  0.64 (0.05 – 8.71)  0.92 (0.07 – 12.55) |
| Immunosuppressive medication | 1.33 (0.99 – 1.80) | 0.87 (0.70 – 1.07) | 1.14 (0.62 – 2.10) |
| C-reactive protein (CRP) at first measurement (per 10 unit increase) | 1.00 (0.99 – 1.02) | 1.01 (1.00 – 1.02) | 1.03 (1.01 – 1.04) |
| Change in CRP levels  Increased/no change  Decreased  Not measured | Ref  1.43 (1.03 – 1.99)  1.02 (0.69 – 1.53) | Ref  1.04 (0.85 – 1.27)  0.77 (0.61 – 0.97) | Ref  1.00 (0.54 – 1.85)  0.67 (0.31 – 1.42) |
| Leukocytes at first measurement | 1.02 (1.00 – 1.03) | 1.00 (0.99 – 1.01) | 1.00 (0.97 – 1.03) |
| Change in leukocyte count  Increased/no change  Decreased  Not measured | Ref  0.95 (0.67 – 1.35)  0.72 (0.47 – 1.10) | Ref  1.17 (0.95 – 1.43)  0.97 (0.75 – 1.24) | Ref  0.76 (0.42 – 1.34)  0.57 (0.28 – 1.17) |
| Early warning score (EWS) during the first 24 hours of hospitalization | 0.96 (0.91 – 1.02) | 1.02 (0.98 – 1.05) | 1.14 (1.02 – 1.27) |
| Highest EWS during the last 24 hours measured | 0.95 (0.87 – 1.03) | 0.98 (0.94 – 1.02) | 0.88 (0.75 – 1.02) |
| Fever during the first 24 hours of hospitalization | 1.39 (1.03 – 1.89) | 1.15 (1.00 – 1.33) | 1.34 (0.92 – 1.94) |
| Absence of fever during the last 24 hours measured | 1.03 (0.71 – 1.49) | 0.80 (0.65 – 0.98) | 0.89 (0.46 – 1.70) |
| Systemic antibiotic use prior to admission | 1.02 (0.83 – 1.26) | 1.12 (0.97 – 1.29) | 1.14 (0.81 – 1.61) |
| Positive blood culture | 1.98 (1.50 – 2.61) | 1.66 (1.30 – 2.13) | 1.28 (0.72 – 2.28) |
| Positive urinary culture | 1.60 (1.29 – 1.99) | NA | NA |
| Positive sputum culture | NA | 1.17 (0.96 – 1.43) | NA |
| Positive wound culture | NA | NA | 1.79 (1.07 – 2.98) |
| Diagnosis  UTI - Catheter  UTI - Systemic symptoms  UTI - Cystitis  UTI - Other | Ref  0.68 (0.48 – 0.99)  5.56 (3.19 – 9.68)  15.10 (9.20 – 24.78) | NA | NA |
| Diagnosis  RTI - HAP  RTI – Aspiration pneumonia  RTI – CAP-s  RTI – CAP-m  Other | NA | Ref  3.49 (2.36 – 5.16)  2.86 (1.94 – 4.22)  2.66 (1.86 – 3.81)  2.62 (1.84 – 3.73) | NA |
| Diagnosis  SSTI – Panaritium/paronychia  SSTI – Cellulitis / erysipelas | NA | NA | Ref  1.15 (0.57 – 2.34) |
| Hospital of admission  Hospital A  Hospital B  Hospital C  Hospital D | Ref  0.64 (0.44 – 0.93)  0.74 (0.51 – 1.08)  0.84 (0.57 – 1.24) | Ref  0.63 (0.49 – 0.80)  0.76 (0.59 – 0.96)  1.30 (1.04 – 1.64) | Ref  0.85 (0.44 – 1.64)  0.75 (0.40 – 1.41)  1.36 (0.76 – 2.45) |

UTI, urinary tract infection; RTI; respiratory tract infection; CAP-m, community-acquired pneumonia mild-to-moderate; CAP-s, community-acquired pneumonia severe; HAP, hospital-acquired pneumonia; PSI, pneumonia severity index; SSTI, skin and soft tissue infection; OR: odds ratio; CI: confidence interval; NA: not applicable

# References

1. Rijksinstituut voor Volksgezondheid en Milieu. WHO-FIC classificaties. Available from: https://class.whofic.nl/. Accessed July 14, 2025.
2. Klinkhamer S, Geerlings SE, Knottnerus BJ, et al. Urineweginfecties. 2025. Available from: https://richtlijnen.nhg.org/. Accessed January 30, 2026.
3. National antibiotic guidelines. Available from: adult.nl.antibiotica.app. Accessed January 30, 2026.
4. Terpstra ML, Geerlings SE, van Nieuwkoop C, et al. Optimization of the antibiotic policy in the Netherlands: SWAB guidelines for antimicrobial therapy of urinary tract infections in adults. 2020. Available from: https://swab.nl/nl/richtlijnen-swab. Accessed January 30, 2026.
5. Sieswerda E, Bax HI, Hoogerwerf JJ, et al. The Dutch Working Party on Antibiotic Policy (SWAB) guideline for empirical antibacterial therapy of sepsis in adults. 2020. Available at: https://swab.nl/nl. Accessed January 30, 2026.
6. van Daalen FV, Boersma WG, van de Garde EMW, et al. Management of Community-Acquired Pneumonia in Adults: the 2024 Practice Guideline from The Dutch Working Party on Antibiotic Policy (SWAB) and Dutch Association of Chest Physicians (NVALT). 2024. Available from: https://swab.nl/nl/richtlijnen-swab. Accessed January 30, 2026.
7. Bons SCS, Bouma M, Draijer LW, et al. Bacteriële huidinfecties. 2024. Available from: https://richtlijnen.nhg.org/. Accessed January 30, 2026.
8. NVDV. Cellulitis-Erysipelas onderste extremiteiten. 2013. Available from: richtlijnendatabase.nl. Accessed January 30, 2026.
